# Digital sleep measures and white matter health in the Framingham Heart Study

**DOI:** 10.37349/emed.2021.00045

**Published:** 2021-06-30

**Authors:** Robert Joseph Thomas, Hyun Kim, Pauline Maillard, Charles S. DeCarli, Eric James Heckman, Cody Karjadi, Ting Fang Alvin Ang, Rhoda Au

**Affiliations:** 1 Department of Medicine, Division of Pulmonary, Critical Care & Sleep Medicine, Beth Israel Deaconess Medical Center, Boston, MA 02215, USA; 2 Department of Anatomy & Neurobiology, and Framingham Heart Study, Boston University School of Medicine, Boston, MA 02118, USA; 3 Department of Neurology, University of California Davis Health, Sacramento, CA 95817, USA; 4 Department of Neurology and Epidemiology, Boston University School of Medicine and Public Health, Boston, MA 02118, USA

**Keywords:** Sleep, cardiopulmonary coupling, hypoxia, white matter, diffusion tensor imaging

## Abstract

**Aim:**

Impaired sleep quality and sleep oxygenation are common sleep pathologies. This study assessed the impact of these abnormalities on white matter (WM) integrity in an epidemiological cohort.

**Methods:**

The target population was the Framingham Heart Study Generation-2/Omni-1 Cohorts. Magnetic resonance imaging (diffusion tensor imaging) was used to assess WM integrity. Wearable digital devices were used to assess sleep quality: the (M1-SleepImage^™^ system) and the Nonin WristOx for nocturnal oxygenation. The M1 device collects trunk actigraphy and the electrocardiogram (ECG); sleep stability indices were computed using cardiopulmonary coupling using the ECG. Two nights of recording were averaged.

**Results:**

Stable sleep was positively associated with WM health. Actigraphic periods of wake during the sleep period were associated with increased mean diffusivity. One marker of sleep fragmentation which covaries with respiratory chemoreflex activation was associated with reduced fractional anisotropy and increased mean diffusivity. Both oxygen desaturation index and oxygen saturation time under 90% were associated with pathological directions of diffusion tensor imaging signals. Gender differences were noted across most variables, with female sex showing the larger and significant impact.

**Conclusions:**

Sleep quality assessed by a novel digital analysis and sleep hypoxia was associated with WM injury, especially in women.

## Introduction

Healthy aging is associated with sleep changes that suggest a weakening of the homeostatic sleep process [1]. The resultant propensity to arousals makes sleep more vulnerable to disruptive influences [2–5]. Polysomnographic changes with aging include reduced total sleep time, increased awakenings and lighter sleep, reduced slow wave sleep, and sleep apnea [1]. Sleep pathology is increasingly recognized as a driver of cognitive impairment, including that associated with aging. Stimuli fragmenting sleep in controlled experimental situations, such as auditory stimuli, reliably induce sleepiness, executive dysfunction, and depressed mood [6–9]. Data support an effect of sleep duration/quality and prospective change in cognition [10–13].

Several changes in sleep associated with neurodegeneration including Alzheimer’s disease (AD) suggest a greatly accelerated aging process. These include a markedly reduced K-complex density (number/minute) [14], a reduction in slow wave sleep, paucity of spindles, reduced rapid eye movement (REM) sleep, increased arousals, and overall sleep fragmentation. Sleep apnea is also common in AD and may amplify both sleep fragmentation and disease progression.

Healthy brain aging is dependent on the integrity of both grey and white matter (WM). Sleep pathology-related mechanisms can cause direct injury to the brain, especially the WM tracts, and increase the risk of “WM dementia” [15–18]. Abnormalities of WM including hyperintensities, infarcts, and abnormal diffusion characteristics are considered contributors to cognitive impairment, both as an independent factor and coexisting with cortically mediated disorders such as AD. Sleep pathology impact on WM may be mediated through activation of inflammation, metabolic dysfunction, nocturnal hypertension and endothelial dysfunction. Short sleep duration has also been associated with worse markers of WM integrity in midlife [19]. There are several reports of severe symptomatic sleep apnea patients presenting to sleep centers who are also described as having substantial WM signal change [20, 21], which can reverse after apnea treatment [22]. Residual sleepiness after apnea treatment has been associated with WM abnormality [23]. As it is well established that there is a substantial variability of symptom severity for any given degree of apnea, i.e., clinically asymptomatic apnea is common in the general population, the relationship of apnea and WM pathology at the population level is less well established.

In this report, using an entirely home-based digital sleep assessment approach, we measured nocturnal oxygenation with a finger pulse oximeter, and defined sleep quality analyzing data from a wearable electrocardiogram (ECG), generating novel measures of cardiopulmonary coupling (CPC). We then tested the hypothesis that sleep quality and sleep hypoxia is associated with WM injury signals as assessed using diffusion tensor imaging (DTI) in the Generation-2/Omni-1 cohort of the Framingham Heart Study (FHS).

## Materials and methods

### Population

The FHS was established in 1948, when 5,209 residents of Framingham, Massachusetts, USA, aged 28 to 62 years, were enrolled in a prospective epidemiologic cohort study. In 1971, an additional 5,124 subjects (offspring of the original cohort subjects and the offspring spouses) were enrolled in the Framingham Offspring Study. The Offspring cohort had cycles of periodic health examination on average every 4 years. The 9^th^ Offspring health exam began in April 2011 and concluded in April of 2014.

In the early 1990s, the need to establish a new group of participants reflecting the increasing diversity of the community was recognized. In 1994, the Omni Cohort 1 of the FHS was initiated. The original Omni cohort consisted of 507 men and women of African-American, Hispanic, Asian, Indian, Pacific Islander and Native American origins, who at the time of enrollment were residents of Framingham and the surrounding towns. The Omni Cohort 1 continues to be examined and followed at the same health examination cycle as the Offspring Cohort. Health exam 4 for Omni Cohort 1 coincided with the dates of the Offspring 9^th^ health exam.

The current study samples are comprised of Offspring/Omni-1 participants in the Wearables Sleep Study who also participated in the health exam 9/4 respectively. A subset of these subjects also underwent both structural brain magnetic resonance imaging (MRI) and DTI between 2009 and 2014 as part of a separate larger study of cognition and brain imaging [24]. Participants with prevalent stroke or dementia at the MRI evaluation were excluded from the Wearables Sleep Study. Demographic characteristics are summarized in Table 1. The Institutional Review Boards at all participating institutions approved this study, and subjects gave written informed consent.

### Sleep data acquisition method

The entire sleep physiology assessment was conducted by mail. Participants who agreed where mailed two sleep quality and sleep oxygenation devices, asked to use both for 2 consecutive nights, and then mailed the devices back. The main measures used were the oxygen saturation time under 90%, and the oxygen 3% desaturation index (ODI, number of 3% desaturations/hour of the recording).

### SleepImage^®^

The SleepImage^®^ System (https://sleepimage.com) was used to collect measures used to compute sleep quality. It is a Health Insurance Portability and Accountability Act (HIPAA)-compatible Cloud Computing system currently hosted in the Amazon Cloud, using small single-sensor recording devices. The analysis is also FDA approved as a stand-alone Software as a Medical Device (SaMD) and can be run on any signal recordings that include an ECG or similar information-content signal including plethysmography (PLETH) and is comparable to a full or limited polysomnography [25]. The SleepImage system allows a review of raw data, to the resolution of individual ECG complexes, snoring bursts, and activity-driven sensor displacements.

### The M1 wearable device (MyCardio, LLC; Broomfield, CO. 80021, USA)

This small wearable recorder (https://sleepimage.com) measures continuous ECG, sampled at 600 Hz, expressed in millivolts, 12-bit quantization, with one adhesive pad under the device and a thin wire across the chest to a second pad. Activity and body position are measured by internal accelerometers and gyroscopes, and snoring is detected by induced vibration. The data is uploaded to the SleepImage website, and automatic analysis generating CPC variables, the sleep spectrogram graphs total sleep time (actigraphic), actigraphic wake (after sleep onset), and transient awakenings (by movement).

The device itself has the following dimensions: height: 79.6 mm, width 48.7 mm, thickness 11.7 mm, weight 20 g, and a storage capacity of 500 Mb. The accelerometer within the device has the following specifications: 12-bit quantization, units are in gravitational acceleration “G” units. The Z channel is sampled at 300 Hz, the Y at 37.5 Hz, and the X channel at 37.5 Hz. Collectively, the X, Y, and Z channels are referred to as the “gravity channels”, and used to compute actigraphy, body position and snore vibrations. The M1 starts recording when the ECG is sensed and stops when the ECG is no longer sensed. Participants were instructed to apply the device when ready to sleep. The movement sensor detects non-respiratory trunk movements to estimate the sleep period (start and stop of sleep) and actigraphic wake episodes when very low frequency coupling (VLFC) periods are associated with actigraphic motion.

### Derivation of CPC sleep metrics from sleep devices

**Figure 1.**
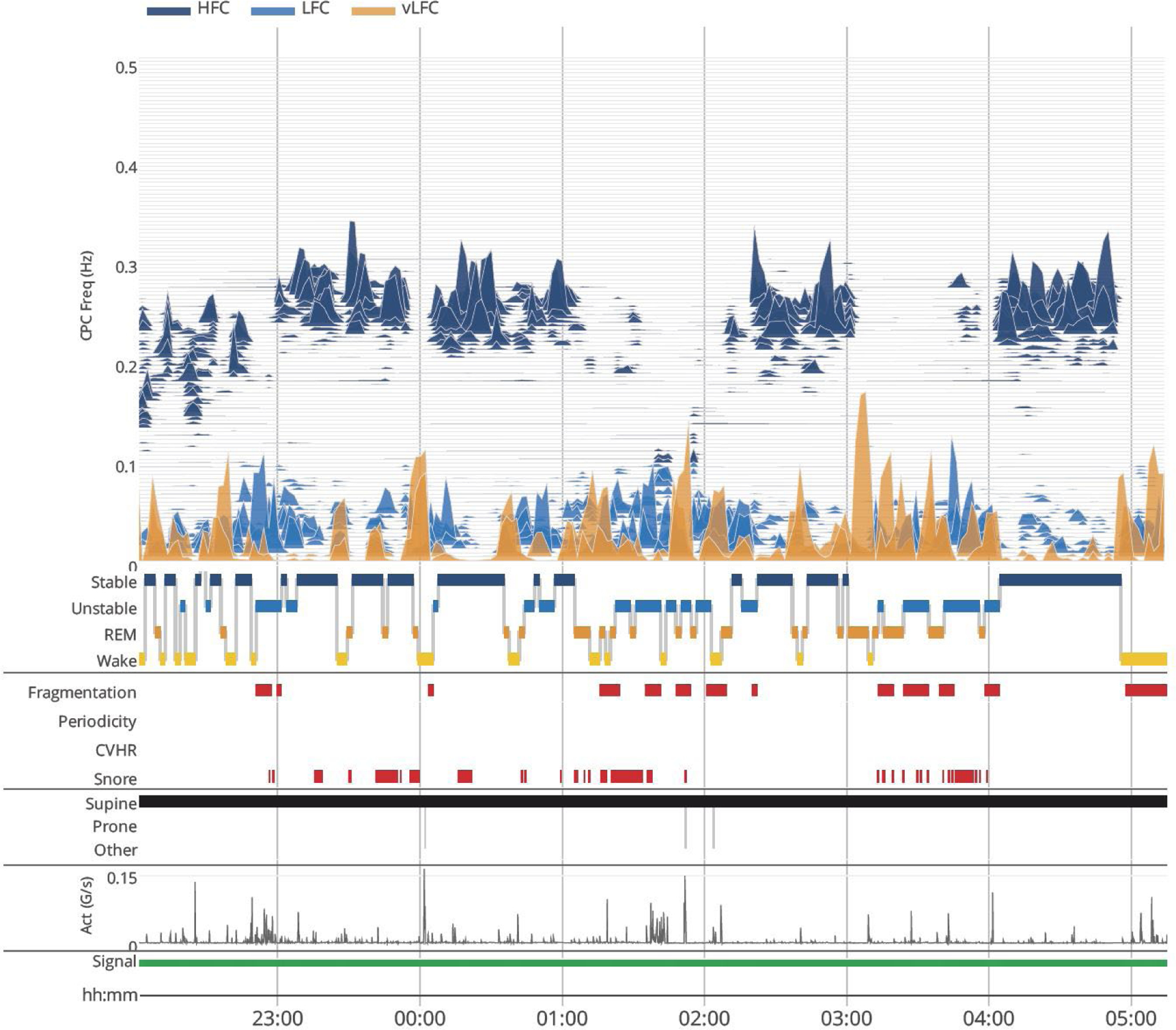
The ECG-spectrogram. Sleep state switches between stable and unstable regimes, high and low frequency coupling (HFC and LFC respectively), respectively. VLFC with movement (actigraphic) is considered wake, while VLFC without movements is considered REM sleep. “fragmentation” is e-LFC and “periodicity” is narrow-band e-LFC

CPC analysis of the ECG signal was performed as previously described [26–28]. The ECG-derived sleep spectrogram [27] uses a time series analysis of the ECG, to generate a measure of “CPC”. Both autonomic drive (through heart rate variability) and respiration (ECG R-wave amplitude fluctuations with individual breaths) are extracted from the continuous ECG and used for analysis. The product of the coherence and cross-spectral power is used to calculate the ratio of coherent cross power in the low frequency (0.01–0.1 Hz) band to that in the high frequency (0.1–0.4 Hz) band. The logarithm of the high to low frequency CPC ratio (log [high frequency coupling (HFC)/low frequency coupling (LFC)]) is then computed to yield a continuously varying measure of CPC. The output is thus a moving average of overlapping CPC windows. The graph of CPC at relevant frequencies (ordinate) *vs*. time (abscissa) provides a sleep spectrogram (Figure 1) The LFC band can be further fractionated, providing a metric, elevated-LFC (e-LFC), which is a measure of sleep fragmentation. This band can be further fractionated into broadband (e-LFC_BB_, generic sleep fragmentation, sleep apnea) or narrowband (e-LFC_NB_), which reflects strong respiratory chemoreflex modulatory effects on sleep state. The relatively slow human heart rate dictates the sampling windows. The analytic window is 8.5 min, and the computation moves over the signal in consecutive 2.1 min windows.

Stable non-rapid eye movement (NREM) sleep is characterized by HFC, increased absolute and relative delta power [28], a consolidated NREM sleep < 1 Hz slow oscillation, temporally stable breathing, stable arousal thresholds, normal arterial oxygen (O_2_) and carbon dioxide (CO_2_) concentrations, and blood pressure (BP) dipping [29]. Unstable NREM is characterized by opposite features. Ineffective (fragmented) REM sleep takes on LFC coupling signatures, while wake or effective REM sleep shows VLFC [27]. An example of the ECG-derived sleep spectrogram is below.

The concept of NREM sleep instability at the electroencephalographic (EEG) level is well established, the cyclic alternating pattern (CAP) [30]. Periods of stable sleep are designated non-CAP. The CPC output correlates poorly with conventional sleep stages, but far more closely with CAP/non-CAP, such that periods of HFC are usually NREM stage N3, but more importantly, the majority of NREM stage N2 in health [27]. LFC aligns with CAP, parts of N2 and light NREM N1 sleep. Disease states expand the proportion of LFC relative to HFC, which are mutually exclusive states.

### MRI including DTI

The dates of MRI evaluation were from 10/13/2011 to 2/28/2018. The scan-sleep measurement time difference mean was −1.42 years (= scan performed about 1.4 years before the sleep study). The longest interval was 3.01 years (= brain scan performed 3 years after the sleep study).

Participants were evaluated with a 1.5-Tesla Siemens Avanto scanner for DTI. The DTI sequence parameters: repetition time (TR) = 3,600 ms, echo time (TE) = 94 ms, 25 contiguous slices total, with no gaps between sections, field-of-view (FOV) = 25 cm, acquisition matrix = 128 × 128, slice thickness = 5 mm. Diffusion weighted images were generated using 30 gradient directions, repeated four times, with total gradient diffusion sensitivity of b = 1,000 s/mm^2^, and four unweighted images with b = 0 s/mm^2^.

DTI images were first preprocessed using FMRIB software library (FSL) software tools [31], including correction for eddy current-induced distortions and participant head movements. Individual fractional anisotropy (FA) maps were coregistered to the FSL FA DTI template using linear and nonlinear transformations. Resulting transformation parameters were applied to the individual FA and mean diffusivity (MD) maps. FSL FA template was thresholded at 0.3 to provide a mask of WM region. For each individual, overall measures of mean FA and MD were computed by superimposing the WM mask onto the respective individual coregistered DTI-derived maps and averaging values within these WM voxels. Peak width of skeletonized MD (PSMD) is a DTI-derived measure based on skeletonization and histogram analysis and is calculated as the difference between the 95^th^ and 5^th^ percentile of MD values within the masked MD skeleton [32].

### Statistical analysis

Both nights of sleep were averaged for analysis. Gender differences in sleep data were analyzed with the *t*-test for normally distributed data and the Mann-Whiney *U* test when variables were skewed. Unadjusted associations between sleep variables (sleep quality, fragmentation, and oxygenation) and DTI measures (FA, MD, PSMD) examined using regression analysis. A multivariate analyses of covariance (MANCOVA) was conducted to test the associations between sleep (HFC, LFC, e-LFC, e-LFC_BB_, e-LFC_NB_, actigraphic wake) and DTI (FA, MD, PSMD) variables, while adjusting for various demographic and clinical covariates (age, sex, body mass index, diabetes, hypertension, hyperlipidemia, and the time interval between polysomnography and DTI). The statistical models were, Model 1: unadjusted; Model 2: adjusted for age and gender; Model 3: adjusted for diabetes, hypertension, hyperlipidemia, body mass index.

Secondary analyses were performed using interaction terms to examine the presence of moderating effect of gender. All analyses were conducted using SAS 9.4 (SAS Institute Inc., Cary, North Carolina).

## Results

### Population characteristics

The demographics of the participants in this analysis are summarized in Table 1. Women were slightly over-represented, and the mean age was 67.92 ± 6.22 years at the time of sleep recording.

### Summary of sleep measures

Sleep measures are summarized in Table 2, including gender differences (Table 3)

### Associations of sleep quality with WM measures

Stable sleep, as identified by the % total sleep time of HFC, was significantly associated with increased FA and reduced MD. Unstable sleep, identified by the % total sleep time of low frequency coupling, was associated only with reduced MD (Table 4). Moderating effects of gender differences were notable with only women showing the impact of protective and injurious effects.

### Association of sleep fragmentation with while matter measures

General sleep fragmentation (e-LFC) was associated in this cross-sectional analysis with increased MD, consistent with increased deterioration of WM integrity. e-LFC_NB_, a signal biomarker of high loop gain (respiratory chemoreflex activation) was significantly associated with reduced FA and increased MD (Table 5). Moderating effects of gender were noted, with only women showing significant effects.

### Associations of sleep hypoxia with WM structure

Both measures of oxygen desaturation had significant cross-sectional associations with DTI measures (Table 6). The oxygen desaturation index was associated with reduced FA and increased MD. The time in minutes under 90% oxygen saturation was associated with reduced FA and increased MD. The peak width of skeletonized MD was positively associated with time under 90% oxygen saturation. Women showed a greater impact on the oxygen desaturation index.

## Discussion

The key outcomes of our analysis were the demonstration that: (1) stable sleep was associated with better WM health (increased FA and reduced MD); (2) actigraphic periods of wake during the sleep period were associated with increased MD; (3) one marker of sleep fragmentation which covaries with respiratory chemoreflex activation was associated with reduced FA and increased MD, DTI markers of deteriorated WM integrity [33–35]; (4) both oxygen desaturation index and oxygen saturation time under 90% were associated with pathological directions of DTI signals. These results were obtained in a community dwelling cohort, rather than a sleep clinic population, and have implications for brain health at the population level [21, 36–39].

Abnormal DTI measures have been associated with a range of conditions with impaired cognition [40–43]. The results of this study are consistent with the concept that sleep pathology including nocturnal hypoxia may accelerate WM injury, and over time, may contribute to vascular mediated AD pathogenesis or vascular dementia. Our results further complement that obtained from studies in clinical sleep apnea populations [21, 44], and reports of increased white matter hyperintensity (WMH) and diffusion tensor abnormalities associated with sleep apnea in a population cohort [37, 45, 46]. As sleep apnea at the population level is often asymptomatic, there may be silent progression of white mater injury which can be additive or synergistic to other brain pathologies.

Gender differences were noted across most variables, with female sex showing the larger and significant impact. Such gender differences of impact of sleep apnea on WM health, with females impacted more severely than males, have been reported [47]. In this study using FA, areas of sex-specific, sleep apnea-related FA reductions appeared in females relative to males, including in the bilateral cingulum bundle adjacent to the mid hippocampus, right stria terminalis near the amygdala, prefrontal and posterior-parietal WM, corpus callosum, and left superior cerebellar peduncle. In this report, females with also showed higher daytime sleepiness, anxiety and depression levels, and reduced sleep quality [47]. There are other reports of gender modifying the impact of sleep pathology on clinical outcomes [48, 49]. Though sleep is deeper by conventional measures and better preserved with age in females, AD is more common in women [50, 51]. Thus, this better sleep does not seem to have neuroprotective effects. Similarly, sleep related complaints, specifically insomnia, is greater in women, again suggesting that sleep quality as currently measured does not reflect underlying biological vulnerability. A study of 122 middle-aged women showed that actigraphic wake after sleep onset was associated with WMHs [52]. The study used wrist actigraphy. Our wake measures used trunk actigraphy but did show MD associated with estimated wake bouts. These two results are thus consistent. Wake events from sleep are associated with substantial transient autonomic activation and surges of BP [53], which may be a mediating mechanism.

The most likely mechanism for the association of sleep pathology with WM injury is dysregulation of BP. The normal drop in BP during restful sleep is reliably lost when sleep is disrupted. This decrease in BP (“dipping”) is a biomarker of health [54], and its absence (“non-dipping”) is associated with a host of poor cardiac, neurological, metabolic and renal outcomes [55–60]. Non-dipping is associated with brain atrophy and cognitive decline [61, 62], and with lower daytime cerebral blood flow [63]. Sleep fragmentation is associated with repetitive BP surges [64, 65] and is associated with daytime hypertension [66]. Sleep deprivation causes mild BP increases [67]. Pathological sleep (sleep apnea, insomnia, restless legs) induces BP non-dipping. Stable sleep as estimated by the CPC method aligns with periods of BP dipping [29]. Thus, nocturnal hypertension is on the mechanistic pathway connecting impaired sleep quality and sleep apnea/nocturnal hypoxia with WM injury.

Our results also show an adverse impact of intermittent nocturnal hypoxia on WM. Exposure to hypoxia has several mechanistic pathways to causing WM injury. Magnetic resonance spectroscopic assessments in severe adult sleep apnea report reduced frontal WM N-acetyl-aspartate and choline [68] and poor post-treatment recovery [69]. Direct effects include free radical mediated injury, lipid peroxidation, induction of nitric oxide synthase, platelet activation factor and apoptosis [70–73]. As the usual cause of intermittent hypoxia is sleep apnea, which is treatable, it will be important to establish if these noted changes are progressive with time, or reversible with treatment.

Sleep quality and fragmentation can impact brain health through multiple mechanisms, including effects on sleep oscillations, sleep state energetics, and glymphatic flow. Stable sleep as measured by HFC has potential protective effects. This biomarker covaries with slow-wave power, an important marker of sleep quality [28]. Of the sleep fragmentation markers, only the one associated with pathological respiratory chemoreflex activation [26] had a statistically significant association with diffusion tensor abnormality. This biomarker, narrowband coupling, is reportedly associated with hypertension and stroke, and increased arousals from sleep, mechanisms which may explain the observed association [74].

Both sleep and WM health evolve with time. One could assume that the level of sleep pathology recorded is likely to have been present for at least 5 years, but there is surprisingly little published data on stability or otherwise of polysomnogram determined sleep at the population level, presumably from the sheer expense associated with such studies. The Sleep Heart Health Study has published the change in respiratory disturbance index over 5 years; the changes are small: the mean respiratory disturbance index increased from 8.1 ± 11 SD at baseline to 10.9 ± 14 [75]. Similarly, the features of WM health slowly evolve slowly over a timescale of years, with an acceleration in the latter decades of life [76–78]. Thus, we have a somewhat broad but relevant overlap of the time scales of sleep pathology and WM health.

The strengths of our study are a well characterized cohort at a vulnerable age where both brain and sleep pathology are common, collection of both sleep quality and sleep oxygenation measures, and sensitive WM assessment with DTI. The limitations of our study include a Caucasian population, modest sample size, non-traditional sleep quality measures even if validated in other conditions, self-selection confounding, and cross-sectional analysis, which can generate hypotheses but not provide further definitive predictive value. The acquisition protocol is relatively low-end: low field (1.5 T) and low resolution (5 mm). The analysis is a whole-brain measure than regional measures, so estimating plausible relationships with neuropsychological sequala is limited.

In summary, we report that sleep quality and sleep oxygenation are associated with WM health as assessed by DTI, with both protective and detrimental effects of sleep stability and sleep hypoxia, respectively, being the most clearly noted. As sleep pathology is highly treatable, and diagnostic assessments increasingly easy and minimally burdensome, targeting sleep to improve brain health could be considered at the population level.

## Figures and Tables

**Table 1.** Demographic information of the study sample

Variable	(Mean ± SD)
Age	67.92 ± 6.22
Sex (female *n*, %)	280 (55.56%)
BMI (kg/m^2^)	28.22 ± 5.22
SBP (mmHg, 1 mmHg = 0.133 kPa)	124.67 ± 15.45
DBP (mmHg)	72.92 ± 9.02
Current smoking	10.00 (1.99%)
Total cholesterol (mg/dL)	185.38 ± 35.39
Total cholesterol ≥ 200 mg/dL	175 (34.72%)
Hypertension treatment	241 (47.91%)
Lipid treatment	175 (34.72%)
Diabetes treatment	41 (8.13%)

BMI: body mass index; SBP: systolic blood pressure; DBP: diastolic blood pressure

**Table 2.** Summary of sleep measures

Sleep variable	(Mean ± SD)
ODI/hour of sleep	7.50 ± 7.26
SpO2 time under 90%	9.39 ± 15.33
HFC duration (min)	191.31 ± 98.97
HFC %	42.00 ± 19.44
LFC duration (min)	171.31 ± 89.92
LFC %	38.15 ± 18.21
e-LFC duration (min)	86.44 ± 68.66
e-LFC %	19.27 ± 14.81
e-LFC_BB_ duration (min)	74.66 ± 56.98
e-LFC_BB_ %	16.66 ± 12.39
e-LFC_NB_ duration (min)	11.78 ± 23.37
e-LFC_NB_ %	2.61 ± 5.07
Wake (#)	40.04 ± 35.19

ODI: oxygen 3% desaturation index; SpO2: pulse oxygen saturation; HFC: high frequency coupling % of total estimated sleep time; LFC: low frequency coupling % of total estimated sleep time; e-LFC: elevated-low frequency coupling % of total estimated sleep time; e-LFC_BB_: broadband e-LFC (generic sleep fragmentation, including apnea); e-LFC_NB_: narrowband e-LFC (activation of the respiratory chemoreflex); Wake: estimated actigraphic wake during the sleep period

**Table 3.** Comparison of sleep variables by gender

Sleep variable	Male (Mean ± SD)	Female (Mean ± SD)	*P*
ODI	9.10 ± 8.80	6.21 ± 5.43	< 0.0001
SpO2	9.57 ± 15.25	9.24 ± 15.42	0.89
HFC duration (min)	152.39 ± 84.53	222.13 ± 98.84	< 0.0001
HFC %	33.75 ± 17.24	48.54 ± 18.60	< 0.0001
LFC duration (min)	201.79 ± 91.83	147.17 ± 80.74	< 0.0001
LFC %	45.44 ± 18.17	32.37 ± 16.08	< 0.0001
e-LFC duration (min)	109.88 ± 77.86	67.88 ± 53.69	< 0.0001
e-LFC %	24.81 ± 16.53	14.89 ± 11.56	< 0.0001
e-LFC_BB_ duration (min)	93.64 ± 64.40	59.63 ± 45.09	< 0.0001
e-LFC_BB_ %	21.16 ± 13.83	13.09 ± 9.76	< 0.0001
e-LFC_NB_ duration (min)	16.24 ± 26.89	8.25 ± 19.49	< 0.0001
e-LFC_NB_ %	3.65 ± 5.81	1.80 ± 4.23	< 0.0001
Wake (#)	44.11 ± 38.91	36.82 ± 31.65	0.02

**Table 4.** Sleep quality and DTI

Diffusion Tensor Measure	Model 1 HFC (*P*)	Model 2 HFC (*P*)	Model 3 HFC (*P*)	Model 1 LFC (*P*)	Model 2 LFC (*P*)	Model 3 LFC (*P*)	Model 1 Wake (*P*)	Model 2 Wake (*P*)	Model 3 Wake (*P*)
FA	0.25	0.03	0.03	0.83	0.11	0.11	0.29	0.12	0.15
MD	0.05	0.01	0.01	0.80	0.23	0.20	0.13	0.01	0.02
PSMD	0.41	0.28	0.26	0.70	0.31	0.25	0.60	0.58	0.86
Moderating effects of gender									
Diffusion Tensor Measure	Model 1 HFC (*P*)	Model 2 HFC (*P*)	Model 3 HFC (*P*)	Model 1 LFC (*P*)	Model 2 LFC (*P*)	Model 3 LFC (*P*)	Model 1 Wake (*P*)	Model 2 Wake (*P*)	Model 3 Wake (*P*)
FA	-	-	0.14	-	-	0.22	-	-	0.22
MD	-	-	0.48	-	-	0.50	-	-	0.15
PSMD	-	-	0.005	-	-	0.02	-	-	0.19

Fully adjusted model shown for gender; statistical significance is shown. Model 1: unadjusted; Model 2: adjusted for age and gender; Model 3: adjusted for diabetes, hypertension, hyperlipidemia, body mass index; -: only full model computed

**Table 5.** Sleep fragmentation and DTI

Diffusion Tensor Measure	Model 1 e-LFC (*P*)	Model 2 e-LFC (*P*)	Model 3 e-LFC (*P*)	Model 1 e-LFC_BB_ (*P*)	Model 2 e-LFC_BB_ (*P*)	Model 3 e-LFC_BB_ (*P*)	Model 1 e-LFC_NB_ (*P*)	Model 2 e-LFC_BB_ (*P*)	Model 3 e-LFC_NB_ (*P*)
FA	0.91	0.04	0.05	0.89	0.15	0.16	0.97	0.03	0.03
MD	1.00	0.06	0.0046	0.99	0.22	0.19	0.97	0.02	0.01
PSMD	0.45	0.45	0.37	0.84	0.82	0.69	0.38	0.11	0.12
Moderating effects of gender									
Diffusion Tensor Measure	Model 1 e-LFC (*P*)	Model 2 e-LFC (*P*)	Model 3 e-LFC (*P*)	Model 1 e-LFC_BB_ (*P*)	Model 2 e-LFC_BB_ (*P*)	Model 3 e-LFC_BB_ ((*P*)	Model 1 e-LFC_NB_ (*P*)	Model 2 e-LFC_BB_ (*P*)	Model 3 e-LFC_NB_ (*P*)
FA	-	-	0.006	-	-	0.15	-	-	< 0.001
MD	-	-	0.06	-	-	0.30	-	-	0.006
PSMD	-	-	0.005	-	-	0.007	-	-	0.15

Fully adjusted model shown for gender; statistical significance is shown; -: only full model computed

**Table 6.** Oxygenation and DTI measures

Diffusion Tensor Measure	Model 1 ODI (*P*)	Model 2 ODI (*P*)	Model 3 ODI (*P*)	Model 1 Time 90 (*P*)	Model 2 Time 90 (*P*)	Model 3 Time 90 (*P*)
FA	0.35	0.02	0.02	0.03	0.02	0.04
MD	0.40	0.01	0.005	0.0002	< 0.001	0.0002
PSMD	0.10	0.01	0.05	0.01	0.007	0.02
Moderating effects of gender						
Diffusion Tensor Measure	Model 1 ODI (*P*)	Model 2 ODI (*P*)	Model 3 ODI (*P*)	Model 1 Time 90 (*P*)	Model 2 Time 90 (*P*)	Model 3 Time 90 (*P*)
FA	-	-	0.02	-	-	0.94
MD	-	-	0.09	-	-	0.85
PSMD	-	-	0.80	-	-	0.22

Fully adjusted model shown for gender; statistical significance is shown; -: only full model computed

## Data Availability

The data supporting this report can be obtained through standard processes from the Framingham Heart Study Service Center. https://framinghamheartstudy.org/fhs-for-researchers/fhs-service-center/

## Abbreviations

AD: Alzheimer’s disease
BP: blood pressure
CAP: cyclic alternating pattern
CPC: cardiopulmonary coupling
DTI: diffusion tensor imaging
ECG: electrocardiogram
e-LFC: elevated-LFC
e-LFCBB: broadband e-LFC
e-LFCNB: narrowband e-LFC
FA: fractional anisotropy
FHS: Framingham Heart Study
FSL: FMRIB software library
HFC: high frequency coupling
LFC: low frequency coupling
MD: mean diffusivity
MRI: magnetic resonance imaging
NREM: non-rapid eye movement
ODI: oxygen 3% desaturation index
PSMD: peak width of skeletonized mean diffusivity
REM: rapid eye movement
SpO2: pulse oxygen saturation
VLFC: very low frequency coupling
WM: white matter
